# BMI and BMI change following incident type 2 diabetes and risk of microvascular and macrovascular complications: the EPIC-Potsdam study

**DOI:** 10.1007/s00125-020-05362-7

**Published:** 2021-01-15

**Authors:** Elli Polemiti, Julia Baudry, Olga Kuxhaus, Susanne Jäger, Manuela M. Bergmann, Cornelia Weikert, Matthias B. Schulze

**Affiliations:** 1 Department of Molecular Epidemiology, German Institute of Human Nutrition Potsdam-Rehbruecke (DIfE), Nuthetal, Germany; 2 German Center for Diabetes Research (DZD), Neuherberg, Germany; 3 Human Study Center, German Institute of Human Nutrition Potsdam-Rehbruecke (DIfE), Nuthetal, Germany; 4 German Federal Institute for Risk Assessment, Department of Food Safety, Berlin, Germany; 5 Institute of Nutritional Science, University of Potsdam, Potsdam, Germany

**Keywords:** BMI, CVD, Diabetes-related vascular complications, Nephropathy, Neuropathy, T2D, Weight change

## Abstract

**Aims/hypothesis:**

Studies suggest decreased mortality risk among people who are overweight or obese compared with individuals with normal weight in type 2 diabetes (obesity paradox). However, the relationship between body weight or weight change and microvascular vs macrovascular complications of type 2 diabetes remains unresolved. We investigated the association between BMI and BMI change with long-term risk of microvascular and macrovascular complications in type 2 diabetes in a prospective cohort study.

**Methods:**

We studied participants with incident type 2 diabetes from the European Prospective Investigation into Cancer and Nutrition (EPIC)-Potsdam cohort, who were free of cancer, cardiovascular disease and microvascular disease at diagnosis (*n* = 1083). Pre-diagnosis BMI and relative annual change between pre- and post-diagnosis BMI were evaluated in multivariable-adjusted Cox models.

**Results:**

There were 85 macrovascular (myocardial infarction and stroke) and 347 microvascular events (kidney disease, neuropathy and retinopathy) over a median follow-up of 10.8 years. Median pre-diagnosis BMI was 29.9 kg/m^2^ (IQR 27.4–33.2), and the median relative annual BMI change was −0.4% (IQR −2.1 to 0.9). Higher pre-diagnosis BMI was positively associated with total microvascular complications (multivariable-adjusted HR per 5 kg/m^2^ [95% CI]: 1.21 [1.07, 1.36], kidney disease 1.39 [1.21, 1.60] and neuropathy 1.12 [0.96, 1.31]) but not with macrovascular complications (HR 1.05 [95% CI 0.81, 1.36]). Analyses according to BMI categories corroborated these findings. Effect modification was not evident by sex, smoking status or age groups. In analyses according to BMI change categories, BMI loss of more than 1% indicated a decreased risk of total microvascular complications (HR 0.62 [95% CI 0.47, 0.80]), kidney disease (HR 0.57 [95% CI 0.40, 0.81]) and neuropathy (HR 0.73 [95% CI 0.52, 1.03]), compared with participants with a stable BMI; no clear association was observed for macrovascular complications (HR 1.04 [95% CI 0.62, 1.74]). The associations between BMI gain compared with stable BMI and diabetes-related vascular complications were less apparent. Associations were consistent across strata of sex, age, pre-diagnosis BMI or medication but appeared to be stronger among never-smokers compared with current or former smokers.

**Conclusions/interpretation:**

Among people with incident type 2 diabetes, pre-diagnosis BMI was positively associated with microvascular complications, while a reduced risk was observed with weight loss when compared with stable weight. The relationships with macrovascular disease were less clear.

**Graphical abstract:**

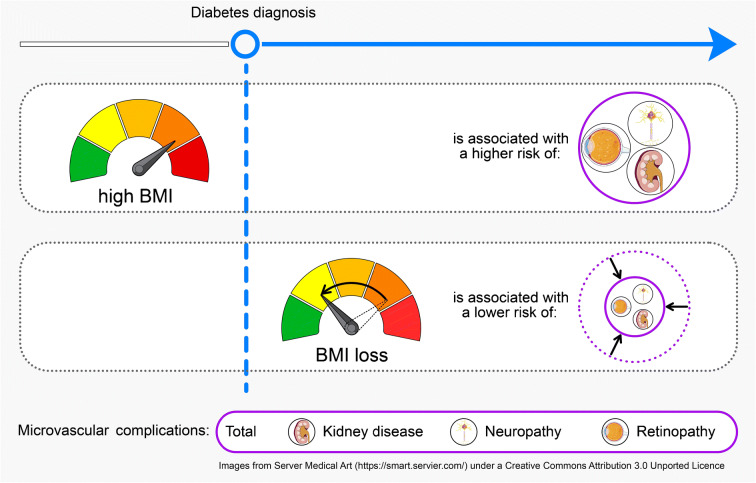

**Supplementary Information:**

The online version of this article (10.1007/s00125-020-05362-7) contains peer-reviewed but unedited supplementary material.

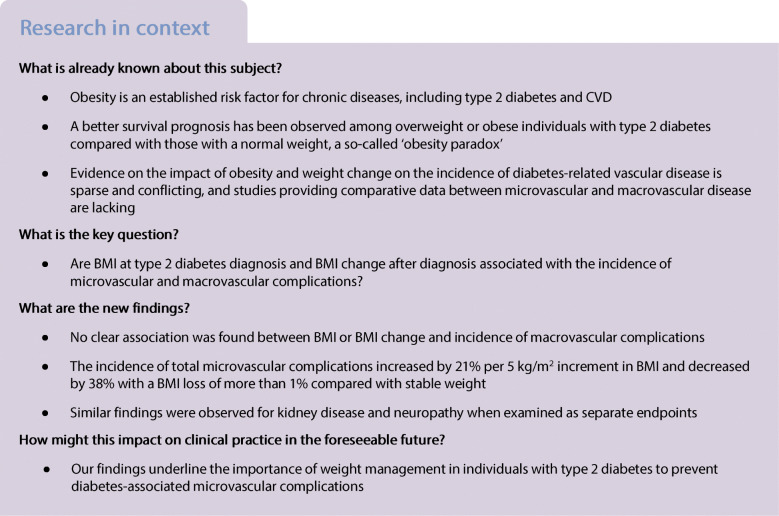



## Introduction

Recent meta-analyses suggest lower all-cause or cardiovascular mortality risk among individuals who are overweight or obese compared with those with normal weight in type 2 diabetes (an ‘obesity paradox’) [1, 2]. These findings, however, might be due to methodological limitations, such as reverse causality, weight effects of pharmacological treatment, short follow-up or suboptimal control for important confounders such as smoking [3].

Longitudinal observational studies investigating the association between obesity and diabetes-related complications have shown inconsistent results. Similar to meta-analyses on cardiovascular mortality, several studies on macrovascular events reported an inverse [4–7] or U-shaped association [8], while others found a positive association [9–11]. Heterogeneous results were also observed for microvascular complications, as positive [11–16], inverse [17, 18] and no associations [19–21] were reported, with the outcome definition varying among studies and the majority examining kidney disease. Altogether, research investigating both microvascular and macrovascular complications is lacking, and most studies are subject to the above-mentioned limitations.

Weight loss after onset of type 2 diabetes may allow remission of diabetes, withdrawal of glucose-lowering drugs [22] and improvement of cardiometabolic risk factors [23]. Nevertheless, weight loss through an intensive lifestyle intervention did not reduce the 10 year CVD risk in the Action for Health in Diabetes (Look AHEAD) trial [24]; only post hoc analyses indicated that a substantial weight loss of at least 10% might reduce risk [25]. Secondary analysis of the Anglo-Danish-Dutch Study of Intensive Treatment in People with Screen-Detected Diabetes in Primary Care (ADDITION-Cambridge) trial observed that ≥5% weight loss decreases 10 year CVD risk [26]. This difference may be explained by the fact that the ADDITION-Cambridge study involved newly diagnosed participants, while Look AHEAD initiated weight loss on average 7 years after diabetes diagnosis. However, secondary analyses of the Diabetes Care in General Practice (DCGP) data in newly diagnosed participants reported that intentional weight loss of 1 kg annually over 6 years was related to an increased non-significant CVD risk [27]. Regarding the relationship between weight change and microvascular complications, data are scarce and rather preliminary. Intentional weight loss might improve renal outcomes in obese individuals with type 2 diabetes but studies are frequently short-term and include individuals with overt kidney disease [28]. Furthermore, lifestyle interventions, including weight loss and exercise, may improve neuropathy symptoms in people with impaired glucose tolerance [29]. Consequently, it becomes apparent that studies investigating weight change are limited or conflicting, and comparative data between microvascular and macrovascular events are lacking.

To address the limitations of previous literature, we investigated the association of BMI and BMI change with long-term risk of both microvascular and macrovascular complications in participants with incident type 2 diabetes from the European Prospective Investigation into Cancer and Nutrition (EPIC)-Potsdam cohort.

## Methods

### Study population

The EPIC-Potsdam study is a population-based prospective cohort study established to investigate the role of diet in chronic disease occurrence. Participants were recruited from 1994 to 1998 in Potsdam, Germany, and the surrounding geographic communities, according to random registry sampling. Overall, 27,548 healthy participants were enrolled, 16,644 women aged 35–64 years and 10,904 men aged 40–64 years [30]. Follow-up questionnaires were implemented every 2–3 years, with response rates exceeding 90% for all follow-up rounds. Detailed information about recruitment and follow-up procedures has been reported elsewhere [30, 31].

Participants with incident type 2 diabetes were identified between recruitment and December 2009 (*n* = 1601; ESM Fig. 1). In May 2014, physicians treating the participants were contacted to obtain information on diabetes-related microvascular and macrovascular complications, extracted from medical records. Individuals without information on vascular complications were excluded from the analysis (*n* = 234). We further excluded participants diagnosed with myocardial infarction, stroke, heart failure, neuropathy, nephropathy, retinopathy or cancer before diabetes diagnosis (*n* = 284), leaving 1083 participants for analysis.

The ethics committee of the Medical Society of the State of Brandenburg, Germany, approved the study protocol. All participants provided written informed consent before enrolment.

### Ascertainment of type 2 diabetes and vascular complications

Follow-up self-report questionnaires were used to identify incident diabetes cases by reporting disease occurrence, disease-relevant medication or dietary treatment. Additional information was obtained from death certificates or clinical record linkage. Physicians treating the participants verified all potential diabetes cases. Only physician-verified diabetes cases (ICD-10 code E11; http://apps.who.int/classifications/icd10/browse/2016/en) with diagnosis date after recruitment were included.

Independently of participants’ vital status, information on incident diabetes-related complications was obtained through standardised forms sent to treating physicians in 2014. The forms collected information related to the latest clinic visit, and occurrence and dates of vascular complications. Incident macrovascular events were also ascertained from the regular follow-up of participants, following the same procedure as diabetes ascertainment.

Microvascular complications comprised diabetic kidney disease (ICD-10 E11.2; unspecified diabetes-related nephropathy, renal replacement therapy or albuminuria), retinopathy (ICD-10 E11.3; proliferative, non-proliferative retinopathy, or blindness) and neuropathy (ICD-10 E11.4; unspecified diabetes-related peripheral neuropathy, amputation, loss of sensation or diabetic foot syndrome).

Macrovascular complications were defined as myocardial infarction (ICD-10 I21) or stroke (ICD-10 I60, I61, I63, I64).

### Assessment of BMI and covariates

At recruitment, weight and height were assessed by trained interviewers following standard protocols [32]. Self-reported weight was obtained through follow-up questionnaires. Pre-diagnosis weight was estimated by the most recent questionnaire before diabetes diagnosis (mean ± SD time, 15 ± 10.8 months). Post-diagnosis weight was identified by the closest questionnaire after diabetes diagnosis (mean ± SD time, 14 ± 9.1 months). BMI was calculated as weight (kg) divided by the square of height (m). Relative annual BMI change was calculated as the difference between post-diagnosis BMI and pre-diagnosis BMI, divided by pre-diagnosis BMI, and further divided by the number of years between the two measurements (mean ± SD time, 2.4 ± 0.55 years) (ESM Fig. 2).

Sociodemographic and lifestyle characteristics were collected using computer-assisted personal interviews at recruitment. These included information on recreational physical activity, dietary habits, alcohol consumption, smoking and education. Dietary and alcohol intake were assessed through validated food frequency questionnaires, as described elsewhere [33]. The MedPyr score was calculated, reflecting adherence to the Mediterranean diet in non-Mediterranean countries [34]. Hypertension was defined at recruitment as systolic BP ≥140 mmHg, diastolic BP ≥90 mmHg, antihypertensive medication use or prior diagnosis of hypertension. Follow-up assessment of hypertension was based on self-reports, and potential cases were verified by treating physicians. Dyslipidaemia was defined at recruitment as lipid-lowering medication use or prior diagnosis of hypertriacylglycerolaemia or hypercholesterolaemia, and during follow-up was retrieved from self-reports. Information on lifestyle factors and health outcomes were updated every 2–3 years or periodically (ESM Table 1). The most recent information before diabetes diagnosis was used. Changes in lifestyle factors were assessed as the difference between post-diagnosis and pre-diagnosis measurements. Diabetes medication data were derived from standardised questionnaires completed by treating physicians during diabetes verification.

### Statistical analysis

Missing values were handled using multiple imputation (*m* = 10) by chained equations [35]. ESM Table 2 provides a summary of missing values and information on multiple imputation method.

BMI was classified according to WHO cut-off points as normal weight (18.5–24.9 kg/m^2^), overweight (25.0–29.9 kg/m^2^), obese I (30.0–34.9 kg/m^2^) and obese II (≥35.0 kg/m^2^) [36]. Relative annual BMI change was divided into three groups: BMI gain (>1%); stable BMI (≤1% gain/loss); and BMI loss (>1%).

We performed separate analyses for total vascular complications, macrovascular complications (MI or stroke), microvascular complications (kidney disease, neuropathy or retinopathy), kidney disease and neuropathy. Analyses for retinopathy, myocardial infarction and stroke as distinct outcomes were not performed due to the limited number of events. Follow-up was defined as the time between diabetes diagnosis and diagnosis of the corresponding vascular disease or date of the last examination by the physicians. The median (IQR) follow-up time was 10.8 (8.2–13.8) years for total complications, 11.6 (9.0–14.6) years for macrovascular complications, 11.1 (8.5–14.0) years for microvascular complications, 11.4 (8.9–14.4) years for kidney disease and 11.4 (9.0–14.4) years for neuropathy. Cox models were performed to estimate HRs for the associations between pre-diagnosis BMI (modelled categorically, with reference group 18.5–24.9 kg/m^2^, and continuously per 5 kg/m^2^ BMI) and incidence of complications, and robust variance estimates were used to calculate 95% CIs [37]. Age was the underlying timescale, with entry time as age at diabetes diagnosis and exit time as age at event or censoring. Three regression models were constructed. The first model was adjusted for age (stratified in years) and sex. The second (main) model was adjusted for age, sex, education (no vocational training/vocational training, technical college degree, university degree), smoking status (never, former, current), smoking duration (years), physical activity (h/week), alcohol consumption (non-drinker [lifetime non-user and former user], very light [men/women ≤2/≤1 g/day], below the limit [men/women >2 to ≤24/>1 to ≤12 g/day], above the limit [men/women >24/>12 g/day]), MedPyr score, and family history of myocardial infarction, stroke and type 2 diabetes. Additional adjustment for prevalent hypertension and dyslipidaemia was performed (model 3). The analysis was performed for all single imputation datasets, and results were combined based on Rubin’s rules [38]. We examined the shape of the associations using restricted cubic splines with knots fitted at the 5th, 50th and 95th percentile of BMI distribution, where median BMI was used as reference. The non-linear trend was tested with the Wald test, and a *p* value <0.05 was considered to indicate a significant non-linear trend.

For analyses of BMI change, participants who developed complications between diabetes diagnosis and post-diagnosis follow-up were excluded (*n* = 11). Follow-up time was defined as above. Secondary analysis with follow-up as the time between post-diagnosis BMI and diagnosis of the corresponding vascular disease or censoring did not alter our results and is not reported. Cox regression and restricted cubic splines were used to estimate HRs for the association between annual BMI change (categorically and per 1%) and incident microvascular and macrovascular complications, with stable BMI and BMI change equal to 0% as references, respectively. The first model included age, sex and pre-diagnosis BMI. The second (main) model included further education, smoking status change (never, former, former-to-current, current-to-former, current), smoking duration, smoking duration change (years), physical activity, physical activity change (h/week), alcohol consumption, alcohol consumption change (g/day), MedPyr score, and antihypertensive (yes/no), lipid-lowering (yes/no) and glucose-lowering medication (no medication, oral medication, insulin, insulin and oral medication).

Consistency of findings was evaluated across strata of sex, age at diabetes diagnosis (<65 vs ≥65 years) and smoking status for pre-diagnosis BMI and BMI change (excluding participants who changed smoking status). For BMI change, we also stratified according to pre-diagnosis BMI (BMI <30.0 vs ≥30.0 kg/m^2^) and oral glucose-lowering medication (yes vs no; excluding insulin users). The likelihood ratio test (LRT) was used to compare models with and without the multiplicative interaction term between continuous BMI and BMI change and the different levels of the effect modifiers. A *p* value of LRT <0.05 was considered significant. We performed sensitivity analyses excluding participants treated with insulin at diagnosis and early outcomes for pre-diagnosis BMI (<2 years after diabetes diagnosis). Early outcomes were not observed for BMI change analysis. To address potential competing risks in participants who developed multiple complications, we repeated the analyses on specific vascular complications, with participants who experienced several events being censored at the first event [39].

Proportional hazards were assessed with Schoenfeld residuals and the linearity of quantitative covariates with cubic splines, as described above. The assumptions of proportional hazards and linearity were fulfilled. Statistical analyses were performed using SAS software, version 9.4, Enterprise Guide 7.1 (SAS Institute, Cary, NC, USA).

## Results

Out of 1083 participants, 587 (54.2%) were men, and there were 85 macrovascular events, 347 total microvascular events, 207 kidney disease events and 211 neuropathy events. Baseline characteristics are presented in Table 1. The median BMI was 29.9 kg/m^2^ (IQR 27.4–33.2). The median age at diabetes diagnosis was 60.4 years (IQR 53.5–65.3) and was lowest in the highest BMI category (57.8 years [IQR 51.2–64.1]). Participants with lower BMI had higher alcohol intake and were more likely to be current smokers and have a university degree. Higher BMI was associated with a higher prevalence of hypertension and family history of diabetes. Median relative annual BMI change after diabetes diagnosis was −0.4% (IQR −2.1 to 0.9), and higher pre-diagnosis BMI was associated with a greater decrease. ESM Tables 3 and 4 report baseline characteristics by sex. Overall, women were diagnosed with diabetes at an older age than men, consumed less alcohol, and were more likely to be never-smokers and to have a family history of diabetes.

**Table 1:** Characteristics of study participants according to pre-diagnosis BMI categories

Characteristic	Total (*n* = 1083)	Pre-diagnosis BMI category			
		18.5–24.9 kg/m^2^ (*n* = 99)	25.0–29.9 kg/m^2^ (*n* = 452)	30.0–34.9 kg/m^2^ (*n* = 377)	≥35.0 kg/m^2^ (*n* = 155)
Pre-diagnosis BMI, kg/m^2^, median (IQR)	29.9 (27.4–33.2)	23.8 (22.8–24.5)	28.0 (26.7–29.1)	32.1 (30.9–33.5)	37.6 (36.1–40.6)
Relative annual BMI change, %, median (IQR)^a,b^	−0.4 (−2.1 to 0.9)	−0.0 (−2.0 to 1.1)	−0.3 (−1.7 to 0.9)	−0.5 (−2.2 to 0.8)	−0.6 (−2.5 to 0.8)
Relative annual BMI change categories, *n* (%)^a^					
> 1% BMI loss	420 (39.3)	37 (37.8)	162 (36.1)	153 (41.2)	69 (45.1)
No change	402 (37.6)	35 (35.7)	179 (40.0)	133 (35.8)	55 (35.9)
> 1% BMI gain	247 (23.1)	26 (26.5)	107 (23.9)	85 (22.9)	29 (19.0)
Demographics					
Male sex, *n* (%)	587 (54.2)	46 (46.5)	268 (59.3)	215 (57.0)	58 (37.4)
Age at pre-diagnosis BMI measurement, years, median (IQR)	59.1 (52.2–64.4)	57.8 (51.0–63.3)	60.2 (54.6–64.8)	58.8 (51.4–64.2)	57.0 (50.3–63.0)
Age at diabetes diagnosis, years, median (IQR)	60.4 (53.5–65.3)	58.9 (52.4–64.5)	61.5 (56.0–66.0)	60.0 (52.3–65.1)	57.8 (51.2–64.1)
Education level, *n* (%)					
No vocational training/vocational training	490 (45.2)	35 (35.4)	187 (41.4)	184 (48.8)	84 (54.2)
Technical college degree	274 (25.3)	28 (28.3)	116 (25.7)	89 (23.6)	40 (25.8)
University degree	320 (29.5)	36 (36.4)	149 (33.0)	104 (27.6)	31 (20.0)
Pre-diagnosis lifestyle					
Physical activity, h/week, median (IQR)	1.0 (0–3.2)	1.0 (0–3.5)	1.0 (0–3.5)	1.0 (0–3.0)	0.6 (0–3.0)
Alcohol intake, g/day, median (IQR)	9.0 (2.8–21.7)	10.1 (2.5–18.6)	10.2 (3.6–22.5)	8.3 (2.9–23.5)	5.8 (1.8–16.3)
MedPyr score, median (IQR)	6.7 (5.8–7.5)	6.7 (5.8–7.5)	6.8 (5.8–7.5)	6.6 (5.8–7.5)	6.8 (5.8–7.7)
Smoking status, *n* (%)					
Never-smoker	428 (39.5)	41 (41.4)	172 (38.1)	147 (39.0)	68 (43.9)
Former smoker	474 (43.7)	35 (35.4)	200 (44.2)	175 (46.4)	65 (41.9)
Current smoker	182 (16.8)	24 (24.2)	80 (17.7)	56 (14.9)	22 (14.2)
Smoking duration, years, median (IQR)	24.0 (15.0–33.0)	24.0 (11.5–34.0)	25.0 (15.0–35.0)	23.0 (15.0–32.3)	26.9 (14.0–31.0)
Medical information, *n* (%)					
Family history of diabetes	483 (44.6)	43 (43.4)	197 (43.6)	169 (44.8)	76 (49.0)
Family history of MI	180 (16.6)	13 (13.1)	78 (17.3)	65 (17.2)	26 (16.8)
Family history of stroke	222 (20.5)	22 (22.2)	99 (21.9)	70 (18.6)	30 (19.4)
Hypertension	870 (80.3)	64 (64.6)	343 (75.9)	317 (84.1)	145 (93.5)
Dyslipidaemia	795 (73.4)	64 (64.6)	347 (76.8)	285 (75.6)	100 (64.5)
Insulin use at diabetes diagnosis	85 (7.8)	9 (9.1)	29 (6.4)	39 (10.3)	7 (4.5)

Table presents combined rounded values from the ten imputation datasets

^a^Fourteen participants did not have follow-up after diabetes diagnosis

^b^Mean ± SD for relative BMI change for the total population was −0.6 ± 2.8

MI, myocardial infarction

### Pre-diagnosis BMI and risk of vascular complications

**Fig. 1:**
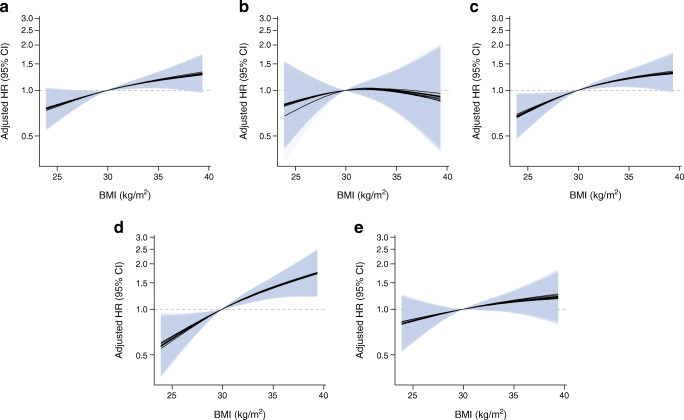
Association between pre-diagnosis BMI and risk of microvascular and macrovascular complications of type 2 diabetes. (**a**) Total vascular complications. (**b**) Macrovascular complications. (**c**) Microvascular complications. (**d**) Kidney disease. (**e**) Neuropathy. Pre-diagnosis BMI was assessed as a continuous variable using restricted cubic spline regression, adjusted for age, sex, education, smoking status, smoking duration, physical activity, alcohol consumption, MedPyr score, family history of diabetes, myocardial infarction and stroke. Splines (black lines) and 95% CIs (blue shading) from ten imputation datasets are shown. Knot placement was 5th, 50th and 95th percentile. Median BMI of 29.9 kg/m^2^ served as reference. Test for non-linearity: total complications, *p* = 0.55; macrovascular complications, *p* = 0.64; microvascular complications, *p* = 0.36; kidney disease, *p* = 0.46; neuropathy, *p* = 0.86

In age- and sex-adjusted Cox regression models, each additional 5 kg/m^2^ higher BMI was associated with 1.17 times higher incidence (95% CI 1.05, 1.30) of total vascular complications (Table 2, model 1). The association did not change substantially in multivariable models further adjusted for education, lifestyle, and family health history (HR 1.18 [95% CI 1.06, 1.31]; Table 2, model 2). Restricted cubic spline analyses did not indicate departure from linearity (*p* for non-linearity = 0.55; Fig. 1). Compared with participants with normal weight, the multivariable-adjusted HR (95% CI) was 1.29 (0.81, 2.04) for participants in the overweight category, 1.57 (0.99, 2.50) for those in the obese I category and 1.97 (1.20, 3.24) for those in the obese II category (Table 2, model 2).

**Table 2:** HRs and 95% CIs for microvascular and macrovascular complications of type 2 diabetes according to pre-diagnosis BMI (categories and per 5 kg/m^2^)

Diabetes complication	BMI category				Continuous BMI, per 5 kg/m^2^ (*n* = 1083)
	18.5–24.9 kg/m^2^ (*n* = 99)	25.0–29.9 kg/m^2^ (*n* = 452)	30.0–34.9 kg/m^2^ (*n* = 377)	≥35.0 kg/m^2^ (*n* = 155)	
Total vascular complications					
No. of cases / person-years	24 / 1073.9	159 / 4844.6	147 / 4047.2	66 / 1663.1	395 / 11,623.9
Age- and sex-adjusted model	1.00 (Ref.)	1.28 (0.81, 2.00)	1.51 (0.97, 2.36)	1.86 (1.15, 3.01)	1.17 (1.05, 1.30)
Model 2^a^	1.00 (Ref.)	1.29 (0.81, 2.04)	1.57 (0.99, 2.50)	1.97 (1.20, 3.24)	1.18 (1.06, 1.31)
Model 3^b^	1.00 (Ref.)	1.23 (0.77, 1.98)	1.51 (0.95, 2.42)	2.05 (1.23, 3.41)	1.19 (1.07, 1.34)
Macrovascular complications					
No. of cases / person-years	7 / 1107.3	38 / 5204.7	32 / 4376.5	8 / 1831.9	85 / 12,516.7
Age- and sex-adjusted model	1.00 (Ref.)	0.91 (0.39, 2.11)	1.02 (0.44, 2.36)	0.70 (0.24, 2.03)	1.01 (0.78, 1.32)
Model 2^a^	1.00 (Ref.)	0.94 (0.40, 2.19)	1.09 (0.45, 2.60)	0.77 (0.26, 2.25)	1.05 (0.81, 1.36)
Model 3^b^	1.00 (Ref.)	0.89 (0.38, 2.11)	0.99 (0.41, 2.40)	0.68 (0.23, 2.04)	1.00 (0.76, 1.31)
Microvascular complications					
No. of cases / person-years	19 / 1108.1	138 / 5082.4	129 / 4218.1	62 / 1707.6	347 / 12,122.7
Age- and sex-adjusted model	1.00 (Ref.)	1.43 (0.86, 2.37)	1.71 (1.04, 2.83)	2.38 (1.39, 4.06)	1.20 (1.07, 1.35)
Model 2^a^	1.00 (Ref.)	1.41 (0.84, 2.37)	1.76 (1.06, 2.95)	2.50 (1.44, 4.36)	1.21 (1.07, 1.36)
Model 3^b^	1.00 (Ref.)	1.33 (0.78, 2.26)	1.69 (1.00, 2.85)	2.70 (1.53, 4.76)	1.24 (1.09, 1.40)
Kidney disease					
No. of cases / person-years	11 / 1130.1	74 / 5309.4	80 / 4386.7	42 / 1778.1	207 / 12,607.7
Age- and sex-adjusted model	1.00 (Ref.)	1.42 (0.73, 2.75)	2.01 (1.04, 3.87)	3.10 (1.57, 6.13)	1.38 (1.20, 1.58)
Model 2^a^	1.00 (Ref.)	1.45 (0.74, 2.82)	2.09 (1.07, 4.07)	3.36 (1.67, 6.79)	1.39 (1.21, 1.60)
Model 3^b^	1.00 (Ref.)	1.37 (0.70, 2.70)	2.02 (1.02, 3.97)	3.57 (1.72, 7.41)	1.42 (1.22, 1.66)
Neuropathy					
No. of cases / person-years	10 / 1118.7	90 / 5250.6	75 / 4417.1	35 / 1794.0	211 / 12,591.1
Age- and sex-adjusted model	1.00 (Ref.)	1.47 (0.75, 2.88)	1.54 (0.79, 2.98)	2.10 (1.03, 4.27)	1.12 (0.96, 1.31)
Model 2^a^	1.00 (Ref.)	1.43 (0.71, 2.87)	1.55 (0.78, 3.10)	2.18 (1.05, 4.54)	1.12 (0.96, 1.31)
Model 3^b^	1.00 (Ref.)	1.32 (0.66, 2.65)	1.41 (0.71, 2.80)	2.14 (1.04, 4.43)	1.11 (0.94, 1.31)

Table presents combined rounded values from the ten imputation datasets

^a^Model 2: age- and sex-adjusted model + education, smoking status, smoking duration, physical activity, alcohol consumption, MedPyr score, family history of diabetes, myocardial infarction and stroke

^b^Model 3: Model 2 + prevalent conditions of hypertension and dyslipidaemia

When evaluating microvascular and macrovascular complications separately, a positive association was observed for microvascular complications (multivariable-adjusted HR per 5 kg/m^2^ BMI increment 1.21 [95% CI 1.07, 1.36]; Table 2, model 2) and no deviation from linearity was found (*p* for non-linearity = 0.36; Fig. 1). The HR (95% CI) was 1.41 (0.84, 2.37) for participants in the overweight category, 1.76 (1.06, 2.95) for those in the obese I category and 2.50 (1.44, 4.36) for those in the obese II category, compared with individuals with normal weight (Table 2, model 2). No association was observed for macrovascular complications (multivariable-adjusted HR per 5 kg/m^2^ BMI increment 1.05 [95% CI 0.81, 1.36]; Table 2, model 2).

Positive linear associations were observed with further subdivision of microvascular complications into kidney disease (multivariable-adjusted HR per 5 kg/m^2^ BMI increment: 1.39 [95% CI 1.21, 1.60]; *p* for non-linearity = 0.46) and neuropathy (HR 1.12 [95% CI 0.96, 1.31]; *p* for non-linearity = 0.86) (Table 2, model 2; Fig. 1). Analyses using BMI categories did not substantially alter the results. Furthermore, the associations between BMI and microvascular complications, neuropathy and kidney disease were robust after further adjustment for dyslipidaemia and hypertension (Table 2, model 3).

Sex-stratified analyses did not reveal substantial differences in associations, except for neuropathy, where a stronger association was present for women (ESM Table 5); nevertheless, this difference was not statistically significant (*p* for LRT = 0.30). In analyses stratified by age at diabetes diagnosis, no substantial differences in associations were detected (ESM Table 6). Among never-smokers, a non-linear association was observed for macrovascular complications (*p* for non-linearity = 0.02), indicating a positive association with a higher BMI up to about 32.5 kg/m^2^ (not shown). Results from sensitivity analyses did not differ substantially from primary analyses (ESM Table 6). Furthermore, no substantial differences were observed when only first-in-order complications were used as endpoints. The corresponding HR (95% CI) per 5 kg/m^2^ BMI increment was 1.03 (0.78, 1.37), 1.21 (1.08, 1.37), 1.39 (1.18, 1.64), 1.07 (0.90, 1.28) for macrovascular complications, microvascular complications, kidney disease and neuropathy, respectively (ESM Table 7, model 2).

### BMI change and risk of vascular complications

In models adjusted for age, sex and pre-diagnosis BMI, BMI loss of >1% per year was associated with a lower hazard of total vascular complications in comparison with stable BMI (HR 0.73 [95% CI 0.58, 0.94]), while no clear association was observed for >1% BMI gain (HR 0.92 [95% CI 0.71, 1.20]) (Table 3, model 1). Further adjustment for lifestyle and medication did not markedly change the results (HR 0.69 [95% CI 0.54, 0.89] for BMI loss; HR 0.86 [95% CI 0.65, 1.14] for BMI gain; Table 3, model 2). A linear trend was observed (*p* for non-linearity = 0.73; ESM Fig. 3) when modelling BMI change per 1% increment, where a positive non-significant association was observed (HR 1.03 [95% CI 0.99, 1.07]; Table 3, model 2).

**Table 3:** HRs and 95% CIs for microvascular and macrovascular complications of type 2 diabetes according to relative BMI change per year (categories and per 1%)

Diabetes complication	BMI change category			Continuous BMI change, per 1% increment (*n* = 1069)
	>1% BMI loss (*n* = 420)	Stable BMI^a^ (*n* = 402)	>1% BMI gain (*n* = 247)	
Total vascular complications				
No. of cases / person-years	141 / 4709.4	158 / 4223.7	82 / 2632.3	380 / 11,568.7
Model 1^b^	0.73 (0.58, 0.94)	1.00 (Ref.)	0.92 (0.71, 1.20)	1.03 (0.99, 1.08)
Model 2^c^	0.69 (0.54, 0.89)	1.00 (Ref.)	0.86 (0.65, 1.14)	1.03 (0.99, 1.07)
Macrovascular complications				
No. of cases / person-years	34 / 4984.6	29 / 4610.2	14 / 2857.9	76/ 12,445.7
Model 1^b^	1.15 (0.69, 1.92)	1.00 (Ref.)	0.80 (0.40, 1.60)	0.93 (0.85, 1.02)
Model 2^c^	1.04 (0.62, 1.74)	1.00 (Ref.)	0.82 (0.42, 1.63)	0.95 (0.87, 1.03)
Microvascular complications				
No. of cases / person-years	120 / 4856.5	147 / 4482.3	75 / 2730.4	341 / 12,068.6
Model 1^b^	0.67 (0.52, 0.86)	1.00 (Ref.)	0.99 (0.75, 1.30)	1.06 (1.02, 1.11)
Model 2^c^	0.62 (0.47, 0.80)	1.00 (Ref.)	0.90 (0.67, 1.21)	1.05 (1.01, 1.10)
Kidney disease				
No. of cases / person-years	66 / 5022.1	88 / 4706.7	48 / 2823.4	202 / 12,551.5
Model 1^b^	0.62 (0.44, 0.86)	1.00 (Ref.)	1.14 (0.80, 1.64)	1.07 (1.01, 1.14)
Model 2^c^	0.57 (0.40, 0.81)	1.00 (Ref.)	1.03 (0.71, 1.50)	1.06 (1.00, 1.13)
Neuropathy				
No. of cases / person-years	74 / 4994.5	94 / 4652.6	42 / 2883.9	209 / 12,530.2
Model 1^b^	0.74 (0.54, 1.03)	1.00 (Ref.)	0.86 (0.60, 1.24)	1.06 (1.01, 1.11)
Model 2^c^	0.73 (0.52, 1.03)	1.00 (Ref.)	0.82 (0.56, 1.20)	1.05 (0.99, 1.11)

Table presents combined rounded values from the ten imputation datasets

Fourteen participants did not have a follow-up after diabetes diagnosis. The number of participants excluded from each model because they developed a complication between diabetes diagnosis and post-diagnosis BMI measurement was as follows: 11 for total complications; 7 for macrovascular complications; 4 for microvascular complications; 3 for kidney disease; 2 for neuropathy

^a^Stable BMI was defined as ≤1% BMI gain/loss

^b^Model 1: adjusted for age, sex and pre-diagnosis BMI

^c^Model 2: Model 1 + education, smoking status change, smoking duration at pre-diagnosis, smoking duration change, physical activity at pre-diagnosis, physical activity change, alcohol consumption at pre-diagnosis, alcohol consumption change, MedPyr score, lipid-lowering medication, antihypertensive medication and glucose-lowering medication

A clearer positive association emerged when microvascular complications were evaluated. The HR (95% CI) for microvascular complications was 1.05 (1.01, 1.10) per 1% increment in BMI change in the final multivariable-adjusted model (Table 3, model 2) and the association was linear (*p* for non-linearity = 0.89; ESM Fig. 3). This finding was corroborated by the categorical analysis where participants with BMI loss were at lower risk of microvascular complications than those with stable BMI. Similarly, a 1% increment in BMI change showed a positive association with both neuropathy (HR 1.05 [95% CI 0.99, 1.11]) and kidney disease (HR 1.06 [95% CI 1.00, 1.13]), and a decreased hazard was observed for BMI loss (Table 3, model 2).

No clear association between BMI change and macrovascular risk was observable. Spline regression, categorical and continuous analyses indicated a modest inverse non-significant association (HR per 1% BMI change 0.95 [95% CI 0.87, 1.03], Table 3, model 2; *p* for non-linearity = 0.37, ESM Fig. 3).

Sex-stratified analyses showed similar associations, except for macrovascular complications, where an inverse association appeared to be present among women, while no meaningful association was observed in men (*p* for LRT <0.001; ESM Table 8). Associations were more prominent for all outcomes among never-smokers (ESM Table 9). There were no substantial differences in associations between BMI change and vascular complications across strata of age at diabetes diagnosis, pre-diagnosis BMI or medication, and in sensitivity analyses excluding insulin users (ESM Table 9), as well as in analyses where only first events were used as final endpoints (ESM Table 10).

## Discussion

Our data revealed a positive association between pre-diagnosis BMI and total vascular complications. The observed association was driven predominantly by microvascular complications, and this applied to both kidney disease and neuropathy. A decreased risk was observed for microvascular complications, kidney disease and neuropathy with BMI loss shortly after diabetes diagnosis. The findings were consistent across different subgroups of sex, age and smoking status for pre-diagnosis BMI, whereas for BMI change the associations were strengthened among never-smokers. In contrast, no apparent association of pre-diagnosis BMI and BMI change with macrovascular complications, comprising myocardial infarction and stroke, was observed.

Previous longitudinal observational studies have shown inconsistent results regarding the association between BMI and microvascular complications. In line with our study, several studies reported a positive association [11–16], whereas others have observed an inverse [17, 18] or no association [19–21]. Of note, only the study by Gray et al. included individuals with newly diagnosed type 2 diabetes [11]. However, this study did not consider comorbid conditions and was based on healthcare claims data, making it susceptible to misclassification and confounding due to inadequate adjustment. Regarding macrovascular events, previous studies showed discordant results by reporting positive [9–11], inverse [4–7] and U-shaped associations [40]. Furthermore, a meta-analysis on cardiovascular mortality found a possible non-linear relationship [1]. Again, reverse causation and confounding by diabetes severity and treatment remains an issue in these studies. Few studies used BMI preceding type 2 diabetes diagnosis as we did in our study. Gray et al. found a positive association between BMI and macrovascular complications [11], whereas two other large cohort studies found inverse associations [4, 5]. Li et al. reported results from several stratified analyses, including smoking status, where the inverse association remained consistent [4]. However, that study was based on data from low-income individuals and lacked information on important lifestyle factors. In contrast, a study in a large US cohort reported a significant positive association between pre-diagnosis BMI and cardiovascular mortality risk among never-smokers with type 2 diabetes [41].

To overcome limitations of previous studies, we used a prospective study, embedded in a population-based cohort, investigating the association of pre-diagnosis BMI with the incidence of both microvascular and macrovascular complications in German individuals with incident type 2 diabetes, while accounting extensively for potential biases. The use of pre-diagnosis BMI protects against misclassification due to weight change by disease severity or medical treatment. Furthermore, excluding participants with pre-existing disease and excluding diabetes-related vascular events during the first years of follow-up prevents reverse causation. In the present study, no obesity paradox was observed. Instead, a clear robust positive association was found with microvascular complications, while BMI was not associated with macrovascular complications.

Several factors could explain the lack of a relationship between obesity and macrovascular complications. First, individuals who are overweight or obese may be treated more intensively for dyslipidaemia, hypertension or hyperglycaemia than counterparts with a normal weight. We do not have measurements of these markers, and therefore we could not assess their changes over time. Adjusting for prevalent hypertension and dyslipidaemia and excluding participants treated with insulin, all representing risk factors for microvascular complications positively associated with BMI, did not change the association. Thus, better treatment of risk factors among obese individuals is unlikely to explain the difference observed for macrovascular vs microvascular complications. Second, sarcopenia may be prevalent among older, leaner people with diabetes, which might predispose to a higher risk of CVD events [42]. Nonetheless, the association did not change after performing a stratified analysis by age at diabetes diagnosis. It is also unlikely that weight loss before diabetes diagnosis could explain these findings as it did not differ meaningfully between BMI categories. Third, suboptimal control for smoking status may lead to spurious results. Restricting our analysis to never-smokers did not change the initial associations for microvascular complications. Yet, the association for macrovascular disease remained uncertain, possibly due to the limited number of macrovascular events.

We evaluated whether BMI change after diabetes diagnosis may influence subsequent vascular complications, given that a weight loss of ≥5% is routinely recommended in individuals who are overweight or obese at type 2 diabetes diagnosis [43]. A limitation of our analyses is that we could not determine whether weight loss was intentional and to what extent weight changes were attributable to different glucose-lowering medications. Still, weight loss was associated with a lower risk of microvascular complications, independent of baseline BMI. In line with our data, a link between intentional weight loss and lower risk of kidney disease in type 2 diabetes has been previously reported [28].

Whether weight loss after diabetes diagnosis is beneficial in terms of CVD risk has been debated. Secondary analysis of the DCGP and Action to Control Cardiovascular Risk in Diabetes (ACCORD) data found that weight loss was linked with a non-significant increase in CVD events [27, 40]. A large Scottish study did not find an association between weight change within 2 years of diabetes diagnosis and 5 year CVD incidence [44], while secondary results of the ADDITION-Cambridge and the Look AHEAD studies observed that weight loss (≥5% and 10%, respectively) decreased risk for CVD incidence at 10 years [25, 26]. We observed an increased risk of macrovascular complications with weight loss, although this association was not significant in the main analysis. Nevertheless, the association was not explained by reverse causation or confounding by smoking in sensitivity analyses.

Our study has several strengths, including the long follow-up and high response in follow-up for complications. Additionally, we performed several stratified and sensitivity analyses to ensure robustness of findings and provided comparative data for microvascular vs macrovascular complications. However, there are limitations. As with all observational studies, we cannot exclude the possibility of residual confounding. Dietary changes were not assessed due to the limited number of repeated measurements. We did not have data on markers of metabolic and cardiovascular health, we had to rely on self-reported weight values, and we did not collect information on other macrovascular complications such as peripheral arterial disease. Microvascular events were recorded by treating physicians and were not monitored during the regular follow-up, which may have resulted in an underestimation of their incidence and prevalence. However, according to the National Disease Management Guidelines [45], individuals with diabetes should be screened for vascular complications once a year, and treating physicians have a central role in managing their care. We are not aware of a national source that provides information on the incidence of diabetes-related complications in Germany for comparison with our data. To account for prevalent vascular complications that were not captured at baseline, we performed analysis excluding early events, where results did not differ substantially. Analysis of fatal cardiovascular events was not possible due to their limited number. Lastly, the population in our study was predominantly white and of higher socioeconomic status, limiting the generalisability of the data.

In conclusion, we found a positive linear association between pre-diagnosis BMI and risk of microvascular complications. Furthermore, weight loss after diagnosis was associated with a decreased risk. The association between BMI and macrovascular disease was less clear. Our study underpins the importance of weight management in preventing major diabetes-associated complications and the need for well-designed studies for macrovascular complications.

## Supplementary Information

ESM (PDF 528 kb)

## Data Availability

The datasets analysed during the current study are not publicly available due to data protection regulations. In accordance with German Federal and State data protection regulations, epidemiological data analyses of EPIC-Potsdam may be initiated upon an informal enquiry addressed to the secretariat of the Human Study Center (Office.HSZ@dife.de). Each request will then have to pass a formal process of application and review by the respective Principal Investigator and a scientific board.

## Abbreviations

ADDITION- Cambridge: Anglo-Danish-Dutch Study of Intensive Treatment in People with Screen-Detected Diabetes in Primary Care
DCGP: Diabetes Care in General Practice
EPIC: European Prospective Investigation into Cancer and Nutrition
Look AHEAD: Action for Health in Diabetes
LRT: Likelihood ratio test
