# Supplementary material for: BMI and BMI change following incident type 2 diabetes and risk of microvascular and macrovascular complications: the EPIC-Potsdam study
Source: Diabetologia. 2021 Jan 15;64(4):814–25. doi: 10.1007/s00125-020-05362-7 (PMC7940263; doi:10.1007/s00125-020-05362-7)
Supplement: Supplementary file 1 — (PDF 528 kb) [file 125_2020_5362_MOESM1_ESM.pdf]

Electronic Supplementary material

BMI and BMI change following incident type 2 diabetes and risk of microvascular and macrovascular complications: the EPIC-Potsdam study

Elli Polemiti; Julia Baudry; Olga Kuxhaus; Susanne Jäger; Manuela M. Bergmann; Cornelia Weikert; Matthias B. Schulze

Supplemental Tables, and Figures are ordered according to their citation in the main manuscript.

Table of Contents

<u>ESM Tables</u>

ESM Table 1 Information collected at recruitment and at each follow-up in EPIC-Potsdam study

ESM Table 2 Summary of missing information

ESM Table 3 Characteristics of study participants according to pre-diagnosis BMI categories, among men

ESM Table 4 Characteristics of study participants according to pre-diagnosis BMI categories, among women

ESM Table 5 HRs and 95% CIs for microvascular and macrovascular complications of diabetes according to pre-diagnosis BMI (per 5 kg/m^2), by sex

ESM Table 6 HRs and 95% CIs for microvascular and macrovascular complications of diabetes according to pre-diagnosis BMI (per 5 kg/m^2), by subgroups and sensitivity analyses

ESM Table 7 HRs and 95% CIs for microvascular and macrovascular complications of diabetes according to pre-diagnosis BMI (categories and per 5 kg/m^2), censored at first event

ESM Table 8 HRs and 95% CIs for microvascular and macrovascular complications of diabetes per 1% relative BMI change per year, by sex

ESM Table 9 HRs and 95% CIs for microvascular and macrovascular complications of diabetes per 1% relative BMI change per year, by subgroups and sensitivity analyses

ESM Table 10 HRs and 95% CIs for microvascular and macrovascular complications of diabetes according to relative BMI change per year (categories and per 1%), censored at first event

ESM Figures

ESM Fig. 1 Flow chart of study sample derivation and number of microvascular and macrovascular complications of diabetes

ESM Fig. 2 Study design

ESM Fig. 3 Association between relative BMI change per year and risk of microvascular and macrovascular complications of diabetes

ESM Tables

ESM Table 1 Information collected at recruitment and at each follow-up in EPIC-Potsdam study

	**Recruitment assessment**	**Follow-up assessment**				
	1994-1998	1st (1997-2001)	2nd (1999-2003)	3rd (2001-2005)	4th (2004-2008)	5th (2007-2009)
**Socio-demographics**	Age, sex, education					
**Lifestyle**	Dietary habits, Physical activity, alcohol consumption, smoking status & duration	Smoking status[a]	Physical activity, alcohol consumption, smoking status & duration	Dietary habits, alcohol consumption, smoking status & duration	Physical activity, smoking status & duration	Physical activity, alcohol consumption, smoking status & duration
**Anthropometry & Medical Information**	Weight, height, waist circumference, medical history, blood pressure	Weight, medical history, medication	Weight, medical history, medication	Weight, medical history, medication	Weight, waist circumference, medical history, medication	Weight, medical history, medication, family history (mother, father, siblings) of MI, stroke or type 2 diabetes

EPIC, European Prospective Investigation into Cancer and Nutrition; MI, Myocardial Infraction. [a]It was based on subsequent follow-up information on smoking status and year of giving up smoking.

ESM Table 2 Summary of missing information[a]

Variable	Missing frequency N (%)
Smoking status, Follow-up 3	1 (0.07)
Smoking status, Follow-up 5	1 (0.08)
Years to heart failure diagnosis[b,c,d]	1 (2.50)
Years to renal replacement therapy[b,d]	1 (25.00)
Smoking status, Follow-up 2[b]	2 (0.15)
Weight, kg, Follow-up 5	2 (0.16)
Waist circumference, cm, EPIC-Potsdam baseline[c]	3 (0.22)
Years to stroke diagnosis[b,d]	3 (3.70)
Years to last examination from treating physician[b]	5 (0.37)
Years to myocardial infarction diagnosis[b,d]	5 (8.93)
Weight, kg, EPIC-Potsdam baseline	6 (0.44)
Height	8 (0.59)
Sports, h/week, Follow-up 4	12 (0.90)
Weight, kg, Follow-up 4	13 (0.98)
Years to retinopathy diagnosis[b,d]	13 (26.53)
Waist circumference, cm, Follow-up 4[c]	14 (1.05)
Weight, kg, Follow-up 2	15 (1.10)
Sports, h/week, Follow-up 2	16 (1.18)
Biking, h/week, Follow-up 2	19 (1.40)
Gardening, h/week, Follow-up 2	19 (1.40)
Alcohol consumption, categories, Follow-up 2	20 (1.47)
Years to nephropathy diagnosis[b,d]	26 (9.59)
Smoking duration, years, EPIC-Potsdam baseline[d]	27 (3.22)
Smoking duration, years, Follow-up 4[d]	27 (3.31)
Years to neuropathy diagnosis[b,d]	28 (10.22)
Smoking duration, years, Follow-up 3[d]	28 (3.39)
Smoking duration, years, Follow-up 5[d]	28 (3.63)
Smoking duration, years, Follow-up 2[d]	29 (3.47)
Smoking status, Follow-up 1	31 (2.28)
Weight, kg, Follow-up 3	43 (3.19)
Alcohol consumption, categories, Follow-up 3	51 (3.79)
Food items required for MedPyr score, Follow-up 3	51 (3.79)
Systolic blood pressure, mmHg, at last examination with treating physicians[c]	56 (4.10)
Diastolic blood pressure, mmHg, at last examination with treating physicians[c]	56 (4.10)
Hemoglobin A1C, %, at last examination with treating physicians[c]	61 (4.46)
Weight, kg, Follow-up 1	65 (4.78)
Sports, h/week, Follow-up 5	75 (5.93)
Serum creatinine, µmol/l, at last examination from treating physicians[c]	89 (6.51)
Family history for type 2 diabetes, myocardial infarction and stroke (parents and siblings)	102 (7.46)
Weight at age 25 years, kg[c]	124 (9.07)
Weight at age 40 years, kg[c]	164 (12.00)

Participants with missing information on the occurrence of outcomes were excluded (n=234). [a]Number of participants for each follow-up of the EPIC-Potsdam study: $N_{EPIC\text{-}Potsdam\ baseline}$=1367; $N_{follow\text{-}up1}$=1,361; $N_{follow\text{-}up2}$=1358; $N_{follow\text{-}up3}$=1346; $N_{follow\text{-}up4}$=1329; $N_{follow\text{-}up5}$=1265. [b]Years to event was calculate as the difference between date of diabetes and subsequent event. [c]Only used in the imputation model. [d]Percentages were calculated among participants with event or behaviour. Missing values were handled using multiple imputation (m=10) by chained equations, with imputation models specified for each variable with missing values separately. The variables were sorted by amount of missing values. Only missing values from completed follow-up rounds were imputed. For continuous variables Box-Cox transformation (for non-normally distributed variables) and the predictive mean matching method were performed. Multiple imputation was performed before the exclusion of prevalent cases. Thus, vascular complications with missing years to event would potentially become prevalent cases. The imputation model included the event indicators, years to event, covariates needed for the analyses model and predictors of incomplete variables.

ESM Table 3 Characteristics of study participants according to pre-diagnosis BMI categories, among men

	Total	Pre-diagnosis BMI category			
	**Total** n=587	**18.5-24.9 kg/m^2** n=46	**25.0-29.9 kg/m^2** n=268	**30.0-34.9 kg/m^2** n=215	**≥35.0 kg/m^2** n=58
Pre-diagnosis BMI, kg/m^2, median (IQR)	29.8 (27.5-32.5)	24.2 (23.3-24.7)	28.1 (26.9-29.1)	32.1 (30.8-33.4)	36.9 (35.8-39.6)
Relative annual BMI change, %, median (IQR)[a]	-0.4 (-1.7 to 0.9)	0.2 (-1.2 to 2.0)	-0.3 (-1.5 to 0.8)	-0.6 (-2.1 to 0.7)	-0.3 (-2.0 to 1.1)
Relative annual BMI change categories, *n* (%)[a]					
>1% BMI loss	222 (37.8)	12 (26.7)	95 (35.8)	92 (43.2)	23 (40.0)
No change	223 (38.0)	16 (35.6)	108 (40.8)	79 (37.1)	21 (36.2)
>1% BMI gain	135 (23.3)	17 (37.8)	62 (23.4)	42 (19.7)	14 (24.1)
Demographics					
Age at pre-diagnosis BMI measurement, years, median (IQR)	58.4 (51.9-63.4)	58.6 (51.6-62.8)	59.6 (54.2-64.2)	56.8 (51.4-63.1)	56.7 (50.7-62.1)
Age at diabetes diagnosis, years, median (IQR)	59.7 (53.1-64.9)	59.6 (52.6-63.7)	60.7 (55.4- 65.3)	57.6 (52.2-64.6)	57.1 (51.0-63.3)
Education, *n* (%)					
No vocational training/vocational training	227 (38.7)	11 (23.9)	102 (38.1)	89 (41.4)	26 (44.8)
Technical college degree	121 (20.6)	11 (23.9)	54 (20.1)	42 (19.5)	14 (24.1)
University degree	239 (40.7)	24 (52.2)	112 (41.8)	84 (39.1)	19 (32.8)
Pre-diagnosis lifestyle					
Physical activity, h/week, median (IQR)	1.0 (0-3.0)	1.0 (0- 3.0)	1.0 (0-3.0)	1.0 (0-3.0)	0.7 (0-3.0)
Alcohol intake, g/day, median (IQR)	16.1 (7.5-33.3)	15.0 (9.9-30.4)	17.0 (7.0-30.0)	16.0 (8.0-36.8)	15.2 (6.3-39.7)
MedPyr score, median (IQR)	6.7 (5.8-7.6)	6.7 (5.7-7.5)	6.8 (5.7-7.5)	6.7 (5.8-7.6)	6.6 (5.9-7.6)
Smoking status, *n* (%)					
Never-smoker	136 (23.2)	10 (21.7)	64 (23.9)	47 (21.9)	15 (25.9)
Former smoker	337 (57.4)	22 (47.8)	149 (55.6)	130 (60.5)	36 (62.1)
Current smoker	114 (19.4)	14 (30.4)	55 (20.5)	38 (17.7)	8 (13.8)
Smoking duration, years, median (IQR)	24.0 (15.0- 33.0)	24.5 (15.0-34.3)	25.0 (15.0-36.0)	23.0 (16.0-32.0)	24.0 (13.5-29.5)
Medical information, *n* (%)					
Family history of diabetes	221 (37.6)	11 (23.9)	100 (37.3)	87 (40.5)	24 (41.4)
Family history of MI	81 (13.8)	4 (8.7)	34 (12.7)	34 (15.8)	9 (15.5)
Family history of stroke	101 (17.2)	8 (17.4)	48 (18.0)	36 (16.7)	9 (15.5)
Hypertension	474 (80.7)	30 (65.2)	208 (77.6)	180 (83.7)	56 (96.6)
Dyslipidaemia	431 (73.4)	29 (63.0)	200 (74.6)	167 (77.7)	34 (58.6)
Insulin use at diabetes diagnosis	49 (8.3)	5 (10.9)	21 (7.8)	21 (9.8)	2 (3.4)

Table presents combined rounded values from the ten imputation datasets. IQR, interquartile range; MI, myocardial infraction. [a]7 participants did not have follow-up after diabetes diagnosis.

ESM Table 4 Characteristics of study participants according to pre-diagnosis BMI categories, among women

	Total	Pre-diagnosis BMI category			
		**18.5-24.9 kg/m²**	**25.0-29.9 kg/m²**	**30.0-34.9 kg/m²**	**≥35.0 kg/m²**
	n=497	n=53	n=185	n=163	n=96
Pre-diagnosis BMI, kg/m², median (IQR)	30.3 (27.2-34.1)	23.7 (22.0-24.2)	27.7 (26.6-29.0)	32.2 (31.1-33.8)	38.3 (36.4-41.2)
Relative annual BMI change, %, median (IQR)[a]	-0.4 (-2.3 to 0.8)	-0.8 (-2.3 to 0.3)	-0.2 (-2.2 to 1.0)	-0.3 (-2.2 to 1.3)	-0.7 (-2.7 to 0.5)
Relative annual BMI change categories, *n* (%)[a]					
>1% BMI loss	199 (40.6)	24 (46.2)	67 (36.8)	62 (38.8)	46 (47.9)
No change	179 (36.5)	19 (36.5)	70 (37.8)	54 (33.8)	35 (36.5)
>1% BMI gain	112 (22.9)	9 (17.3)	45 (24.3)	44 (27.5)	15 (15.6)
Demographics					
Age at pre-diagnosis BMI measurement, years, median (IQR)	60.1 (52.3-65.0)	57.5 (50.1-63.4)	61.6 (55.2-65.4)	60.8 (52.4-65.2)	57.9 (50.3-63.2)
Age at diabetes diagnosis, years, median (IQR)	61.2 (54.0-66.3)	58.5 (50.9-64.7)	63.1 (56.6-67.2)	61.7 (54.2-66.6)	58.8 (51.4-64.6)
Education, *n* (%)					
No vocational training/vocational training	263 (52.9)	24 (45.3)	86 (46.5)	95 (58.3)	58 (60.4)
Technical college degree	153 (30.8)	17 (32.1)	64 (34.6)	48 (29.4)	26 (27.1)
University degree	81 (16.3)	12 (22.6)	37 (20.0)	20 (12.3)	12 (12.5)
Pre-diagnosis lifestyle					
Physical activity, h/week, median (IQR)	1.0 (0-3.5)	1.0 (0-4.0)	1.0 (0-3.5)	1.0 (0-3.6)	0.6 (0-2.6)
Alcohol intake, g/day, median (IQR)	4.3 (1.5-9.4)	5.5 (1.7-12.0)	5.5 (1.6-10.8)	3.3 (1.6-7.1)	2.9 (1.4-7.5)
MedPyr score, median (IQR)	6.7 (5.8-7.5)	6.9 (5.9-7.6)	6.7 (6.0-7.5)	6.5 (5.8-7.3)	7.0 (5.8-7.7)
Smoking status, *n* (%)					
Never-smoker	292 (58.8)	31 (58.5)	108 (58.4)	100 (61.3)	53 (55.2)
Former smoker	137 (27.6)	12 (22.6)	51 (27.6)	44 (27.0)	29 (30.2)
Current smoker	68 (13.7)	10 (18.9)	25 (13.5)	18 (11.0)	15 (15.6)
Smoking duration, years, median (IQR)	24.0 (12.6-33.0)	20.8 (4.0-31.8)	24.0 (13.5-33.5)	20.5 (12.0-33.5)	27.5 (15.5-32.5)
Medical information, *n* (%)					
Family history of diabetes	262 (52.7)	32 (60.4)	96 (51.9)	83 (50.9)	53 (55.2)
Family history of MI	100 (20.1)	9 (17.0)	43 (23.2)	30 (18.4)	18 (18.8)
Family history of stroke	121 (24.3)	14 (26.4)	51 (27.6)	35 (21.5)	22 (22.9)
Hypertension	396 (79.7)	34 (64.2)	135 (73.0)	137 (84.0)	90 (93.8)
Dyslipidaemia	365 (73.4)	35 (66.0)	148 (80.0)	118 (72.4)	65 (67.7)
Insulin use at diabetes diagnosis	36 (7.2)	4 (7.5)	8 (4.3)	18 (11.0)	5 (5.2)

Table presents combined rounded values from the ten imputation datasets. IQR, interquartile range; MI, myocardial infraction. [a]7 participants did not have follow-up after diabetes diagnosis.

ESM Table 5 HRs and 95% CIs for microvascular and macrovascular complications of diabetes according to pre-diagnosis BMI (per 5 kg/m^2), by sex

	Men	Women
**Total complications**		
No. of cases / person-years	241 / 6354.6	155 / 5270.4
Age-adjusted model	1.14 (0.99, 1.32)	1.18 (0.99, 1.40)
Model 2	1.17 (1.00, 1.36)	1.21 (1.00, 1.45)
Model 3	1.17 (1.00, 1.38)	1.19 (0.99, 1.43)
**Macrovascular complications**		
No. of cases / person-years	57 / 6878.4	28 / 5638.8
Age-adjusted model	1.05 (0.75, 1.49)	0.93 (0.60, 1.45)
Model 2	1.06 (0.74, 1.51)	1.02 (0.63, 1.67)
Model 3	1.01 (0.69, 1.46)	0.93 (0.53, 1.62)
**Microvascular complications**		
No. of cases / person-years	206 / 6579.4	142 / 5389.3
Age-adjusted model	1.17 (1.00, 1.38)	1.22 (1.02, 1.46)
Model 2	1.22 (1.02, 1.45)	1.24 (1.02, 1.51)
Model 3	1.24 (1.03, 1.5)	1.22 (1.00, 1.50)
**Kidney disease**		
No. of cases / person-years	124 / 6864.6	83 / 5589.2
Age-adjusted model	1.38 (1.12, 1.69)	1.32 (1.05, 1.64)
Model 2	1.50 (1.21, 1.87)	1.32 (1.01, 1.73)
Model 3	1.54 (1.22, 1.94)	1.30 (0.98, 1.71)
**Neuropathy**		
No. of cases / person-years	119 / 6868.6	92 / 5570.4
Age-adjusted model	0.99 (0.79, 1.24)	1.23 (0.98, 1.56)
Model 2	1.03 (0.81, 1.31)	1.22 (0.96, 1.54)
Model 3	1.00 (0.77, 1.29)	1.20 (0.95, 1.52)

Table presents combined rounded values from the ten imputation datasets. Model 2: age-adjusted model + education, smoking status, smoking duration, physical activity, alcohol consumption, MedPyr score, family history of diabetes, MI and stroke; Model 3: Model 2 + prevalent conditions of hypertension and dyslipidaemia. Likelihood ratio test for interaction term: Total complications: p=0.96; Macrovascular complications: p=0.48; Microvascular complications: p=0.86; Kidney disease: p=0.64; Neuropathy: p=0.30.

ESM Table 6 HRs and 95% CIs for microvascular and macrovascular complications of diabetes according to pre-diagnosis BMI (per 5 kg/m^2), by subgroups and sensitivity analyses

	Total complications	Macrovascular	Microvascular	Kidney disease	Neuropathy
**Excluding early outcomes**					
No. of cases / person-years	369 / 11,595.3	70 / 12,504.8	333 / 11,953.9	199 / 12,599.7	205 / 12,581.7
Age- and sex- adjusted model	1.17 (1.05, 1.31)	1.01 (0.76, 1.36)	1.20 (1.06, 1.35)	1.38 (1.20, 1.59)	1.11 (0.94, 1.30)
Model 2	1.19 (1.06, 1.32)	1.05 (0.79, 1.40)	1.21 (1.07, 1.37)	1.40 (1.21, 1.61)	1.12 (0.96, 1.32)
**Excluding insulin users**					
No. of cases / person-years	351 / 10,816.9	77 / 11,601.6	306 / 11,273.3	186 / 11,685.0	181 / 11,693.7
Age- and sex- adjusted model	1.14 (1.02, 1.28)	1.03 (0.78, 1.37)	1.18 (1.04, 1.33)	1.29 (1.11, 1.49)	1.09 (0.93, 1.28)
Model 2	1.15 (1.03, 1.29)	1.07 (0.81, 1.42)	1.18 (1.04, 1.34)	1.30 (1.11, 1.52)	1.10 (0.94, 1.30)
**Never smoker**					
No. of cases / person-years	136 / 4671.6	22 / 4982.3	127 / 4801.0	77 / 4955.4	77 / 4983.5
Age- and sex- adjusted model	1.19 (0.97, 1.45)	-[a]	1.17 (0.95, 1.44)	1.24 (0.96, 1.60)	1.16 (0.88, 1.53)
Model 2	1.20 (0.98, 1.46)	-[a]	1.16 (0.94, 1.43)	1.20 (0.93, 1.58)	1.12 (0.84, 1.49)
**Former smoker**					
No. of cases / person-years	185 / 5157.3	42 / 5584.1	157 / 5399.1	98 / 5624.8	94 / 5626.7
Age- and sex- adjusted model	1.21 (1.02, 1.43)	-[a]	1.29 (1.08, 1.55)	1.51 (1.24, 1.85)	1.16 (0.92, 1.47)
Model 2	1.20 (1.00, 1.44)	-[a]	1.31 (1.07, 1.61)	1.64 (1.30, 2.06)	1.18 (0.91, 1.52)
**Current smoker**					
No. of cases / person-years	75 / 1794.6	21 / 1953.2	65 / 1920.6	32 / 2026.4	41 / 1980.5
Age- and sex- adjusted model	1.10 (0.85, 1.42)	1.21 (0.75, 1.97)	1.08 (0.81, 1.45)	1.52 (1.05, 2.20)	1.04 (0.72, 1.50)
Model 2	1.21 (0.87, 1.67)	1.38 (0.57, 3.31)	1.19 (0.85, 1.66)	1.77 (1.08, 2.90)	1.31 (0.81, 2.13)
**Age at diagnosis <65y**					
No. of cases / person-years	276 / 8960.6	51 / 9612.6	247 / 9294.9	140 / 9671.8	155 / 9608.6
Age- and sex- adjusted model	1.19 (1.05, 1.34)	1.00 (0.72, 1.37)	1.20 (1.05, 1.38)	1.47 (1.25, 1.74)	1.07 (0.89, 1.29)
Model 2	1.18 (1.05, 1.33)	1.03 (0.74, 1.44)	1.20 (1.04, 1.37)	1.50 (1.26, 1.79)	1.06 (0.89, 1.27)
**Age at diagnosis ≥65y**					
No. of cases / person-years	119 / 2664.9	34 / 2904.0	101 / 2828.2	67 / 2935.0	57 / 2982.5
Age- and sex- adjusted model	1.14 (0.91, 1.43)	1.05 (0.65, 1.69)	1.23 (0.97, 1.55)	1.18 (0.91, 1.55)	1.32 (0.98, 1.78)
Model 2	1.17 (0.92, 1.48)	1.14 (0.68, 1.89)	1.25 (0.97, 1.61)	1.23 (0.91, 1.67)	1.42 (1.02, 1.99)

Table presents combined rounded values from the ten imputation datasets. Model 2: age- and sex-adjusted model + education, smoking status, smoking duration, physical activity, alcohol consumption, MedPyr score, family history of diabetes, MI and stroke. [a]HR (95% CI) is not reported where violation of linearity assumption was found. Likelihood ratio test for interaction term for smoking status: Total complications: p=0.76; Microvascular complications: p=0.72; Microvascular complications: p=0.48; Kidney disease: p=0.24; Neuropathy: p=0.99. Likelihood ratio test for interaction term for age at diabetes diagnosis: Total complications: p=0.36; Macrovascular complications: p=0.74; Microvascular complications: p=0.82; Kidney disease: p=0.93; Neuropathy: p=0.93.

ESM Table 7 HRs and 95% CIs for microvascular and macrovascular complications of diabetes according to pre-diagnosis BMI (categories and per 5 kg/m^2), censored at first event

	BMI category				Continuous BMI,
	**18.5-24.9 kg/m^2**	**25.0-29.9 kg/m^2**	**30.0-34.9 kg/m^2**	**≥35.0 kg/m^2**	**per 5 kg/m^2**
	n=99	n=452	n=377	n=155	n=1083
**Macrovascular complications**					
No. of cases / person-years	6 / 1073.9	34 / 4844.6	26 / 4047.2	8 / 1663.1	74 / 11,623.9
Age- and sex-adjusted model	1.00 (Ref.)	0.95 (0.39, 2.30)	0.97 (0.40, 2.39)	0.82 (0.27, 2.47)	1.03 (0.78, 1.37)
Model 2	1.00 (Ref.)	0.99 (0.41, 2.42)	1.00 (0.39, 2.53)	0.86 (0.29, 2.61)	1.03 (0.78, 1.37)
Model 3	1.00 (Ref.)	0.94 (0.38, 2.32)	0.90 (0.35, 2.32)	0.75 (0.24, 2.33)	0.97 (0.72, 1.31)
**Microvascular complications**					
No. of cases / person-years	18 / 1073.9	125 / 4844.6	121 / 4047.2	58 / 1663.1	321 / 11,623.9
Age- and sex-adjusted model	1.00 (Ref.)	1.39 (0.82, 2.34)	1.70 (1.01, 2.86)	2.26 (1.30, 3.93)	1.20 (1.07, 1.36)
Model 2	1.00 (Ref.)	1.39 (0.81, 2.38)	1.76 (1.03, 3.02)	2.42 (1.36, 4.31)	1.21 (1.08, 1.37)
Model 3	1.00 (Ref.)	1.32 (0.76, 2.29)	1.71 (0.98, 2.96)	2.68 (1.48, 4.83)	1.26 (1.11, 1.42)
**Kidney disease**					
No. of cases / person-years	8 / 1073.9	56 / 4844.6	63 / 4047.2	31 / 1663.1	157 / 11,623.9
Age- and sex-adjusted model	1.00 (Ref.)	1.52 (0.72, 3.19)	2.11 (1.01, 4.41)	2.92 (1.3, 6.54)	1.36 (1.16, 1.60)
Model 2	1.00 (Ref.)	1.63 (0.76, 3.48)	2.36 (1.10, 5.06)	3.44 (1.47, 8.03)	1.39 (1.18, 1.64)
Model 3	1.00 (Ref.)	1.55 (0.71, 3.38)	2.26 (1.04, 4.92)	3.68 (1.52, 8.90)	1.43 (1.20, 1.69)
**Neuropathy**					
No. of cases / person-years	9 / 1073.9	67 / 4844.6	54 / 4047.2	24 / 1663.1	154 / 11,623.9
Age- and sex-adjusted model	1.00 (Ref.)	1.34 (0.64, 2.79)	1.48 (0.72, 3.05)	1.76 (0.80, 3.87)	1.07 (0.90, 1.27)
Model 2	1.00 (Ref.)	1.29 (0.60, 2.81)	1.49 (0.69, 3.19)	1.79 (0.79, 4.10)	1.07 (0.90, 1.28)
Model 3	1.00 (Ref.)	1.22 (0.56, 2.66)	1.43 (0.67, 3.07)	1.95 (0.86, 4.43)	1.09 (0.91, 1.31)

Table presents combined rounded values from the ten imputation datasets. Model 2: age- and sex-adjusted model + education, smoking status, smoking duration, physical activity, alcohol consumption, MedPyr score, family history of diabetes, myocardial infarction and stroke; Model 3: Model 2 + prevalent conditions of hypertension and dyslipidaemia

ESM Table 8 HRs and 95% CIs for microvascular and macrovascular complications of diabetes per 1% relative BMI change per year, by sex

	Men	Women
**Total complications**		
No. of cases / person-years	235 / 6319.0	145 / 5249.3
Model 1	1.03 (0.98, 1.09)	1.04 (0.97, 1.12)
Model 2	1.03 (0.97, 1.08)	1.05 (0.98, 1.13)
**Macrovascular complications**		
No. of cases / person-years	55 / 6841.7	22 / 5606.8
Model 1	0.96 (0.85, 1.07)	0.86 (0.74, 0.99)
Model 2	0.98 (0.87, 1.11)	0.85 (0.73, 1.00)
**Microvascular complications**		
No. of cases / person-years	203 / 6644.4	139 / 5423.3
Model 1	1.06 (1.01, 1.12)	1.07 (0.99, 1.16)
Model 2	1.05 (0.99, 1.12)	1.06 (0.98, 1.15)
**Kidney disease**		
No. of cases / person-years	122 / 6925.9	80 / 5624.8
Model 1	1.07 (1.00, 1.16)	1.06 (0.95, 1.18)
Model 2	1.07 (0.98, 1.15)	1.04 (0.93, 1.16)
**Neuropathy**		
No. of cases / person-years	118 / 6936.1	91 / 5590.7
Model 1	1.07 (1.00, 1.14)	1.09 (1.00, 1.18)
Model 2	1.06 (0.99, 1.14)	1.09 (0.99, 1.19)

Table presents combined rounded values from the ten imputation datasets. Model 1: adjusted for age and pre-diagnosis BMI; Model 2: Model 1 + education, smoking status change, smoking duration at pre-diagnosis, smoking duration change, physical activity at pre-diagnosis, physical activity change, alcohol consumption at pre-diagnosis, alcohol consumption change, MedPyr score, lipid-lowering medication, antihypertensive medication, glucose-lowering medication. For models of total complications 4 men/ 6 women were excluded because they developed a complication between diabetes diagnosis and next follow-up; for macrovascular complications 1 man/ 5 women; for microvascular complications 3men/ 1 woman; for kidney disease 2men/ 1woman; for neuropathy 1man/ 1 woman. Likelihood ratio test for interaction term: Total complications: p=0.78; Macrovascular complications: p<0.001; Microvascular complications: p=0.53; Kidney disease: p=0.64; Neuropathy: p=0.13.

ESM Table 9 HRs and 95% CIs for microvascular and macrovascular complications of diabetes per 1% relative BMI change per year, by subgroups and sensitivity analyses

	Total complications	Macrovascular	Microvascular	Kidney disease	Neuropathy
**Excluding insulin users**					
No. of cases / person-years	338 / 10,769.5	69 / 11,538.6	300 / 11,230.5	182 / 11,637.7	180 / 11,641.0
Model 1	1.03 (0.98, 1.07)	0.92 (0.83, 1.01)	1.06 (1.01, 1.11)	1.07 (1.00, 1.14)	1.05 (1.00, 1.11)
Model 2	1.02 (0.98, 1.06)	0.94 (0.86, 1.03)	1.05 (1.00, 1.10)	1.07 (1.00, 1.14)	1.04 (0.98, 1.10)
**Never smoker[a]**					
No. of cases / person-years	128 / 4650.0	18 / 4652.1	123 / 4734.8	73 / 4889.7	76 / 4906.1
Model 1	1.10 (1.01, 1.19)	0.84 (0.68, 1.03)	1.16 (1.07, 1.26)	1.15 (1.04, 1.28)	1.16 (1.04, 1.29)
Model 2	1.09 (1.01, 1.18)	0.78 (0.62, 0.97)	1.15 (1.06, 1.25)	1.13 (1.02, 1.25)	1.16 (1.04, 1.29)
**Former smoker[a]**					
No. of cases / person-years	174 / 5032.2	38 / 5033.6	149 / 5373.0	93 / 5415.8	91 / 5419.4
Model 1	1.01 (0.96, 1.07)	0.96 (0.84, 1.10)	1.03 (0.97, 1.10)	1.10 (1.00, 1.22)	1.02 (0.94, 1.10)
Model 2	1.00 (0.94, 1.07)	0.94 (0.83, 1.06)	1.02 (0.95, 1.09)	1.08 (0.98, 1.20)	1.01 (0.93, 1.11)
**Current smoker[a]**					
No. of cases / person-years	58 / 1787.1	19 / 1369.7	49 / 1465.4	23 / 1543.7	33 / 1507.5
Model 1	0.91 (0.78, 1.06)	1.05 (0.84, 1.32)	0.86 (0.69, 1.06)	0.85 (0.68, 1.06)	0.86 (0.66, 1.12)
Model 2	0.89 (0.74, 1.07)	-[b]	0.78 (0.61, 1.01)	-[b]	-[b]
**Age at diagnosis <65y**					
No. of cases / person-years	271 / 8949.8	49 / 9605.6	244 / 9285.7	138 / 9664.4	153 / 9603.9
Model 1	1.02 (0.97, 1.07)	0.87 (0.80, 0.95)	1.06 (1.01, 1.11)	1.05 (0.98, 1.13)	1.06 (1.00, 1.12)
Model 2	1.03 (0.95, 1.12)	0.89 (0.81, 0.98)	1.05 (1.00, 1.11)	1.04 (0.97, 1.12)	1.05 (0.98, 1.11)
**Age at diagnosis ≥65y**					
No. of cases / person-years	109 / 2617.3	28 / 2840.0	97 / 2784.3	64 / 2887.1	56 / 2926.6
Model 1	1.08 (1.00, 1.16)	1.08 (0.91, 1.27)	1.07 (0.99, 1.17)	1.13 (1.01, 1.26)	1.06 (0.97, 1.16)
Model 2	1.02 (0.94, 1.11)	0.99 (0.84, 1.16)	1.03 (0.94, 1.13)	1.08 (0.95, 1.23)	1.11 (1.00, 1.22)
**Pre-diagnosis BMI <30.0 kg/m^2**					
No. of cases / person-years	176 / 5899.7	40 / 6290.1	156 / 6182.0	83 / 6428.7	101 / 6352.6
Model 1	1.05 (0.99, 1.12)	0.96 (0.83, 1.12)	1.09 (1.02, 1.16)	1.14 (1.04, 1.25)	1.06 (0.99, 1.14)
Model 2	1.05 (0.98, 1.12)	0.97 (0.86, 1.11)	1.09 (1.01, 1.17)	1.15 (1.03, 1.29)	1.06 (0.98, 1.14)

Continued

ESM Table 9 continued

	Total complications	Macrovascular	Microvascular	Kidney disease	Neuropathy
**Pre-diagnosis BMI ≥30.0 kg/m^2**					
No. of cases / person-years	204 / 5665.7	37 / 6154.7	185 / 5884.6	118 / 6117.0	108 / 6167.9
Model 1	1.03 (0.98, 1.09)	0.90 (0.82, 0.99)	1.06 (1.00, 1.13)	1.04 (0.96, 1.12)	1.11 (1.02, 1.20)
Model 2	1.02 (0.97, 1.08)	0.95 (0.84, 1.06)	1.05 (0.99, 1.11)	1.04 (0.96, 1.12)	1.08 (0.99, 1.17)
**No glucose-lowering medication at diagnosis**					
No. of cases / person-years	96 / 3616.2	16 / 3843.2	85 / 3780.9	56 / 3895.5	45 / 3894.5
Model 1	1.05 (0.96, 1.14)	0.85 (0.68, 1.05)	1.09 (0.99, 1.20)	1.12 (0.97, 1.29)	-[c]
Model 2	1.06 (0.98, 1.16)	0.74 (0.55, 0.99)	1.12 (1.02, 1.23)	1.13 (0.99, 1.29)	-[c]
**Glucose-lowering medication at diagnosis[d]**					
No. of cases / person-years	242 / 7156.0	53 / 7697.6	216 / 7450.4	126 / 7742.9	135 / 7746.5
Model 1	1.00 (0.95, 1.06)	0.94 (0.84, 1.05)	1.03 (0.97, 1.09)	1.05 (0.98, 1.13)	1.04 (0.97, 1.11)
Model 2	1.00 (0.95, 1.06)	0.95 (0.86, 1.05)	1.02 (0.96, 1.08)	1.05 (0.98, 1.14)	1.03 (0.96, 1.11)

Table presents combined rounded values from the ten imputation datasets. Model 1: adjusted for age, sex and BMI at diabetes diagnosis; Model 2: Model 1 + education, smoking status change, smoking duration at pre-diagnosis, smoking duration change, physical activity at pre-diagnosis, physical activity change, alcohol consumption at pre-diagnosis, alcohol consumption change, MedPyr score, lipid-lowering medication, antihypertensive medication, glucose-lowering medication. [a]Participants who changed smoking status were excluded. [b]HR (95% CI) is not reported due to sparse data. [c]HR (95% CI) is not reported where violation of linearity assumption was found. [d]Insulin users were excluded.

Likelihood ratio test for interaction for smoking status: Total complications: *p*=0.09; Macrovascular complications: *p*=0.74; Microvascular complications: *p*=0.02; Kidney disease: p=0.34; Neuropathy: *p*=0.04. Likelihood ratio test for interaction for age at diabetes diagnosis: Total complications: *p*=0.04; Macrovascular complications: *p*=0.35; Microvascular complications: *p*=0.11; Kidney disease: *p*=0.001; Neuropathy: *p*=0.75. Likelihood ratio test for interaction term for pre-diagnosis BMI: Total complications: p=0.44; Macrovascular complications: p=0.67; Microvascular complications: p=0.05; Kidney disease: p=0.03; Neuropathy: p=0.83. Likelihood ratio test for interaction term for glucose-lowering medication at diabetes diagnosis: Total complications: p=0.27; Macrovascular complications: p=0.53; Microvascular complications: p=0.26; Kidney disease: p=0.28; Neuropathy: p=0.86.

ESM Table 10 HRs and 95% CIs for microvascular and macrovascular complications of diabetes according to relative BMI change per year (categories and per 1%), censored at first event

	BMI change category			Continuous BMI change, per 1% increment
	**>1% BMI loss**	**Stable BMI[a]**	**>1% BMI gain**	
	n=420	n=402	n=247	n=1069
**Macrovascular complications**				
No. of cases / person-years	31 / 4709.4	23 / 4223.7	12 / 2632.3	66 / 11,568.7
Model 1	1.27 (0.72, 2.23)	1.00 (Ref.)	0.81 (0.38, 1.70)	0.92 (0.83, 1.02)
Model 2	1.23 (0.69, 2.21)	1.00 (Ref.)	0.84 (0.40, 1.77)	0.94 (0.85, 1.03)
**Microvascular complications**				
No. of cases / person-years	110 / 4709.4	134 / 4223.7	70 / 2632.3	314 / 11,568.7
Model 1	0.65 (0.50, 0.84)	1.00 (Ref.)	0.95 (0.71, 1.26)	1.06 (1.02, 1.11)
Model 2	0.61 (0.46, 0.80)	1.00 (Ref.)	0.87 (0.64, 1.18)	1.05 (1.00, 1.10)
**Kidney disease**				
No. of cases / person-years	49 / 4709.4	67 / 4223.7	37 / 2632.3	153 / 11,568.7
Model 1	0.56 (0.38, 0.82)	1.00 (Ref.)	1.00 (0.65, 1.52)	1.06 (0.99, 1.14)
Model 2	0.52 (0.35, 0.79)	1.00 (Ref.)	0.91 (0.58, 1.42)	1.05 (0.97, 1.13)
**Neuropathy**				
No. of cases / person-years	57 / 4709.4	66 / 4223.7	29 / 2632.3	152 / 11,568.7
Model 1	0.72 (0.49, 1.05)	1.00 (Ref.)	0.80 (0.52, 1.22)	1.05 (0.99, 1.11)
Model 2	0.69 (0.47, 1.01)	1.00 (Ref.)	0.77 (0.49, 1.20)	1.04 (0.98, 1.11)

Table presents combined rounded values from the ten imputation datasets. Model 1: adjusted for age, sex and pre-diagnosis BMI; Model 2: Model 1 + education, smoking status change, smoking duration at pre-diagnosis, smoking duration change, physical activity at pre-diagnosis, physical activity change, alcohol consumption at pre-diagnosis, alcohol consumption change, MedPyr score, lipid-lowering medication, antihypertensive medication, glucose-lowering medication. [a]Stable BMI was defined as ≤1% BMI gain/loss. 14 participants did not have a follow-up after diabetes diagnosis. For models of total complications 11 participants were further excluded because they developed a complication between diabetes diagnosis and post-diagnosis BMI measurement; for macrovascular complications 7 participants; for microvascular complications 4 participants; for kidney disease 3 participants; for neuropathy 2 participants.

ESM Figures

ESM Fig. 1 Flow chart of study sample derivation and number of microvascular and macrovascular complications of diabetes

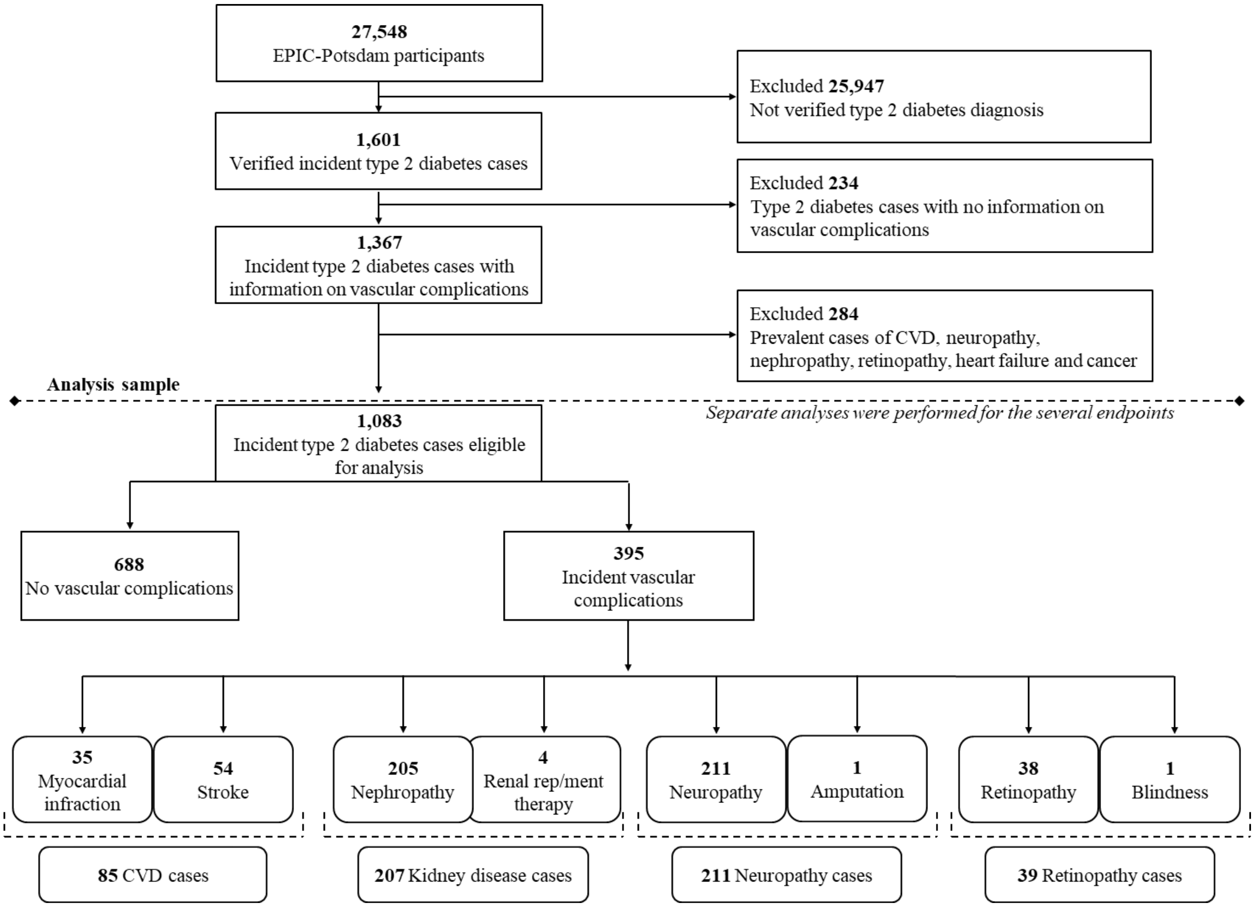

EPIC, European Prospective Investigation into Cancer and Nutrition; CVD, Cardiovascular Disease

Incident macrovascular complications (CVD), n=85

Incident microvascular complications (kidney disease, neuropathy, retinopathy), n=347

Participants with both macrovascular and microvascular complications, n=37; Participants with one complication, n=271; Participants with two complications, n=104; Participants with three complications, n=20.

ESM Fig. 2 Study design

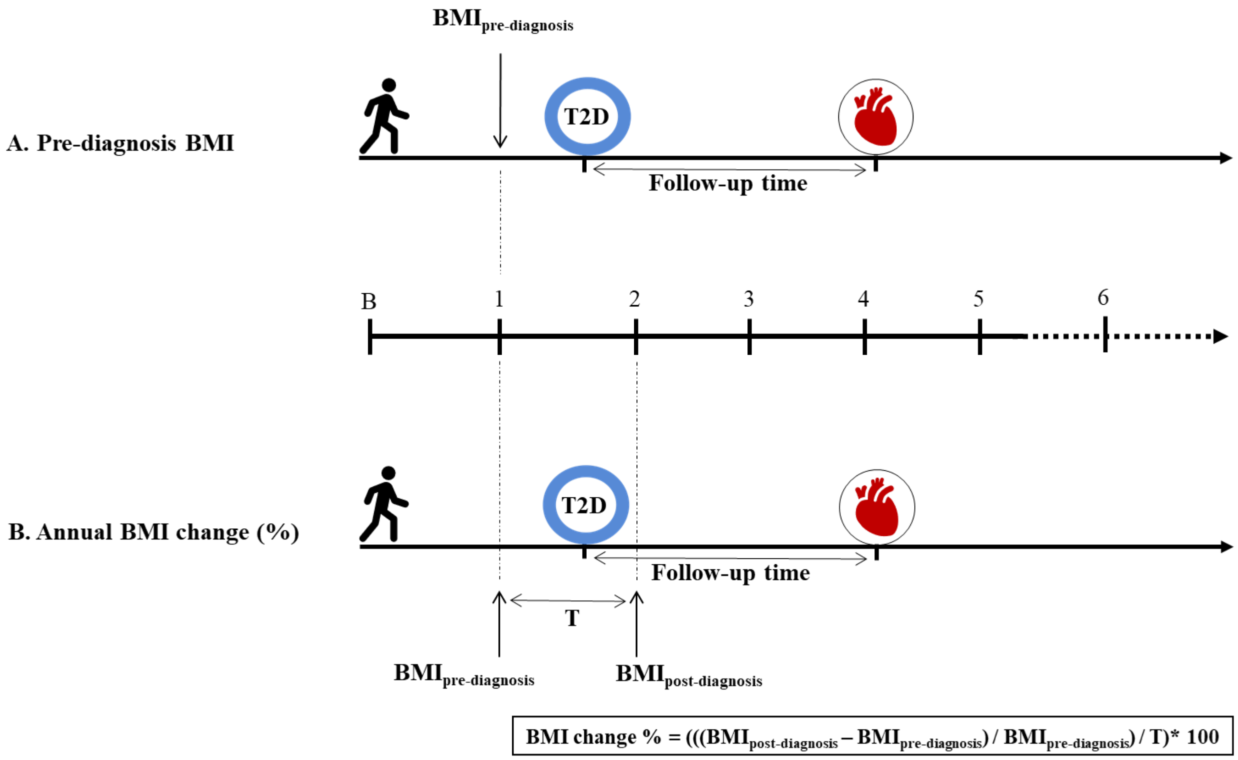

The line in the middle represents the timeline of the European Prospective Investigation into Cancer and Nutrition (EPIC)-Potsdam study, where: B=Baseline, 1 to 6=Follow-up round 1 to 6. The figure visualises the design of the current study, presenting the example of one participant who developed type 2 diabetes between follow-up 1 and 2 of the EPIC-Potsdam study, and was diagnosed with a macrovascular complication at around follow-up 4. For the present study, the follow-up time was defined as the time between type 2 diabetes diagnosis and diagnosis of the corresponding vascular disease or date of the last examination by the physicians. For the analysis on annual BMI change, participants who developed complications between diabetes diagnosis and post-diagnosis BMI measurement were excluded (n=11).

ESM Fig. 3 Association between relative BMI change per year and risk of microvascular and macrovascular complications of diabetes. (**a**) Total vascular complications. (**b**) Macrovascular complications. (**c**) Microvascular complications. (**d**) Kidney disease. (**e**) Neuropathy.

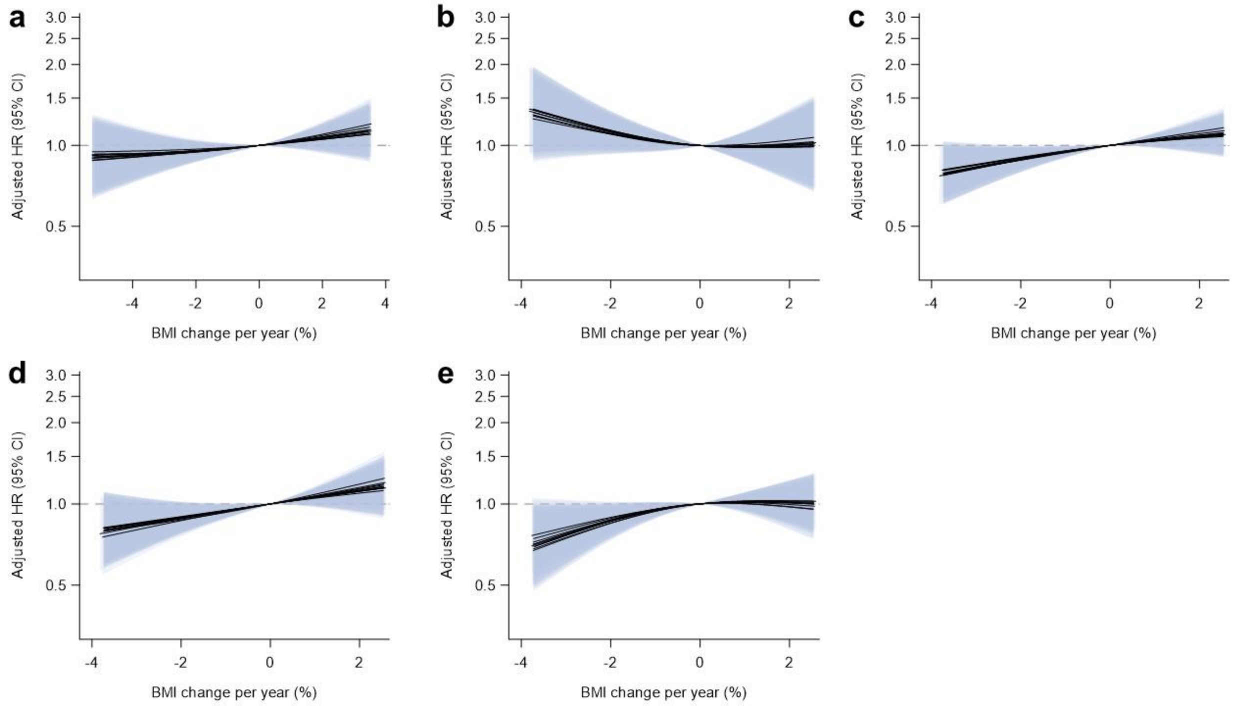

Relative annual BMI change was assessed as a continuous variable using restricted cubic spline regression, adjusted for age, sex, BMI at diabetes diagnosis, education, smoking status change, smoking duration at pre-diagnosis, smoking duration change, physical activity at pre-diagnosis, physical activity change, alcohol consumption at pre-diagnosis, alcohol consumption change, MedPyr score, lipid-lowering medication, antihypertensive medication, glucose-lowering medication. Splines (black lines) and 95% CIs (blue shading) from ten imputation datasets are shown. Knot placement was 5th, 50th and 95th percentile. BMI change of 0% served as reference. Test for nonlinearity: Total complications, p=0.73; Macrovascular complications, p=0.37; Microvascular complications, p=0.89; Kidney disease, p=0.66; Neuropathy, p=0.18.
